# Phase Stability and Compressibility of 3R-MoN_2_ at High Pressure

**DOI:** 10.1038/s41598-019-46822-4

**Published:** 2019-07-19

**Authors:** Xuefeng Zhou, Mingqi Yan, Mingdong Dong, Dejiang Ma, Xiaohui Yu, Jianzhong Zhang, Yusheng Zhao, Shanmin Wang

**Affiliations:** 1 Department of Physics, Southern University of Science & Technology, Shenzhen, 518055 China; 2 Institute of Physics, Chinese Academy of Sciences, Beijing, 100190 China; 3 Materials Science & Technology Division, Los Alamos National Laboratory, Los Alamos, NM 87545 USA

**Keywords:** Electronic properties and materials, Electronic devices

## Abstract

We report phase stability and compressibility of rhombohedral 3R-MoN_2_, a newly discovered layer-structured dinitride, using *in-situ* synchrotron high-pressure x-ray diffraction measurements. The obtained bulk modulus for 3R-MoN_2_ is 77 (6) GPa, comparable with that of typical transition-metal disulfides (such as MoS_2_). The axial compressibility along *a* axis is more than five times stiffer than that along *c* axis. Such strong elastic anisotropy is mainly attributed to its layered structure with loosely bonded N-Mo-N sandwich interlayers held by weak Van der Waals force. Upon compression up to ~15 GPa, a new hexagonal phase of 2H-MoN_2_ occurs, which is irreversible at ambient conditions. The structural transition mechanism between 3R and 2H phases is tentatively proposed to be associated with the rotation and translation of sandwich interlayers, giving rise to different layer stacking sequences in both phases. At high temperature, the decomposition of 3R-MoN_2_ leads to the formation of hexagonal *δ-*MoN and the onset degassing temperature increases as the pressure increases. In addition, the low-temperature electrical resistivity measurement indicates that 3R-MoN_2_ behaves as a semiconductor with an estimated band gap of E_g_ ≈ 0.5 eV. 3R-MoN_2_ also shows weak antiferromagnetic properties, which probably originates from the occurrence of magnetic zigzag edges in the structure.

## Introduction

Transition-metal (TM) nitrides are a class of technologically important compounds and have attracted considerable attention because they exhibit many unique properties and can be used as hard protective coatings (e.g., TiN and CrN)^1^, semiconductors (ScN)^2^, superconductors (*e.g*., NbN)^3^, and superior catalysts^4,5^. Among TM nitrides, molybdenum nitrides (Mo-N) often exhibit interest properties particularly^4–10^. As a typical example, hexagonal δ-MoN holds the highest hardness in the family of metal nitrides with the second highest superconducting temperature (*i.e*., T_c_ ≈14 K)^11^. Thus, the search for new nitrides in the Mo-N system will provide great opportunities for fundamental studies and industrial applications in many fields of science and technology.

However, synthesis of these nitrides is still challenging because the incorporation of nitrogen into the crystalline lattices of transition metals is thermodynamically unfavorable at atmospheric pressure. As a result, most of the reported TM nitrides are poorly crystallized and nitrogen-deficient with molar ratios of N: metal less than unity, which severely limits their use in diverse technological applications. In the binary Mo-N system, three different phases with varying nitrogen concentrations have been reported, referring to ref.^5^ for a thorough overview of this system. In spite of the fact that an oxidation state of Mo as high as +6 occurs in other chemical systems (*e.g*., MoO_3_), the synthesis of nitrogen-rich nitride, MoN_2_, is still limited by traditional synthetic routes at ambient pressure.

Thanks to recent advancements in high-pressure techniques, a number of novel nitrogen-rich TM nitrides have recently been synthesized from direct metal-gas (N_2_) reactions in a pressure range of 18–50 GPa^12^. The new compounds include *Th*_3_*P*_*4-*_type A_3_N_4_ (A = Zr and Hf)^13^ and noble metal dinitrides (OsN_2_, IrN_2_, and PtN_2_)^14,15^. Successful high-*P* synthesis of nitrides with higher oxidation states demonstrates that pressure can effectively promote the role of *d*-electrons in chemical bonding with nitrogen. Most recently, we have successfully synthesized a series of novel nitrogen-rich tungsten nitrides (*e.g*., W_2_N_3_ and W_3_N_4_) through a newly formulated solid-state ion-exchange reactions between Na_2_*X*O_4_ (*X* = Cr, Mo and W) and *h*BN at pressures up to 5 GPa, which is in the pressure range of the current technological capability for massive, industrial-scale production^16,17^. Of particular interest is the discovery of a novel nitrogen-rich nitride, 3R-MoN_2_ using this formulated synthesis methodology. Strikingly, the new nitride is explored to adopt a rhombohedral MoS_2_-type structure (*i.e*., a layered structure), which consist typically of one plane of hexagonally packed metal atoms sandwiched by two planes of nitrogen atoms. The sandwich layers are vertically stacked and loosely bonded by weak van der Waals forces as suggested by theoretical simulations^9,18^, similar to that in the TM dichalcogenides^19^. Besides, our preliminary experiments indicate that 3R-MoN_2_ demonstrates highly catalytic activities for hydrogenation processes, and it may hold great promise as the next-generation catalysts and energy storage materials for a wide range of applications^7,8^. Regarding physical properties of 3R-MoN_2_, to date, it has sparsely been explored, but store exciting physics, especially for the material has the form of an atomic-level thin MoN_2_ sheet. Remarkably, recent theoretical calculations indicate that the monolayer MoN_2_ may have intriguing structural, electronic, and magnetic properties^6,9,18^.

For the layer-structured material systems, it often exists a series of different polymorphs such as hexagonal 2H and rhombohedral 3R polytypes as demonstrated in MoS_2_^20^. The only structural difference between 2H and 3R phases is their stacking sequences of close-packed sandwich layers. Because of the weak interlayer interaction, both polymorphs can readily be converted between them through the interlayer rotation coupled with translation at certain high pressure and temperature^20^. Apparently, it provides an effective protocol to prepare new polytype 2H-MoN_2_ by treating the synthesized 3R-MoN_2_ under high pressure conditions. To the best of our knowledge, in addition to its electronic and magnetic properties, the phase stability of the newly synthesized 3R-MoN_2_ has not yet been investigated which further limits its industrial applications, calling for more experimental data on this material.

With these aims in this work, we present a comprehensive study on 3R-MoN_2_ with focus on the phase stability and compressibility using high-*P* synchrotron XRD measurements, leading to the discovery of a new 2H-MoN_2_. The elastic, electronic, and magnetic properties of 3R-MoN_2_ have also been explored in detail.

## Experimental Section

High-purity Na_2_MoO_4_ (>99.5%, ~50 μm) and *h*BN (>99.9%, ~50 μm) powders in the molar ratio Na_2_MoO_4_: BN = 1: 2 were homogeneously mixed for the synthesis of the nitride. High *P-T* synthesis experiments were performed using a DS 6 × 14 MN cubic press and the detailed experimental procedures have previously been described in refs^5,21^. The run products were washed with distilled water to remove the byproduct NaBO_2_ and unreacted Na_2_MoO_4_, followed by drying in an oven at 348 K. To obtain phase-pure nitride, a two-step reaction route was adopted, referring to ref.^5^ for more experimental descriptions.

High-*P* angle-dispersive synchrotron x-ray diffraction (XRD) experiments using a diamond-anvil cell (DAC) were performed up to 30 GPa at the HPCAT 16BM-D beamline of the Advanced Photon Source (APS). In each of the high-P experiment, the nitride powders with submicron grain size were loaded into the sample hole in a stainless-steel gasket pre-indented to ~30 microns in thickness with neon as the pressure-transmitting medium. A few ruby balls were also loaded into the sample hole to serve as the internal pressure standard. High *P-T* energy-dispersive synchrotron diffraction experiments were performed up to 10 GPa and 1273 K in a large-volume high-*P* apparatus installed at the X17B2 beamline of the National Synchrotron Light Source (NSLS). The experimental details for angle- and energy-dispersive synchrotron measurements have been described previously^16,22^. The crystal structure was determined from analysis of the x-ray data using the GSAS software^23^.

Low-*T* magnetic susceptibility and four-probe resistivity measurements were conducted on a bulk sample sintered at 3.5 GPa and 753 K for 8 hours to measure the electric and magnetic properties. The final bulk sample was 4 mm in diameter and 1 mm in thickness. The density of the sintered sample was measured using the Archimedes method, and the obtained value is within more than 90% of the x-ray determined density.

## Results and Discussion

**Figure 1:**
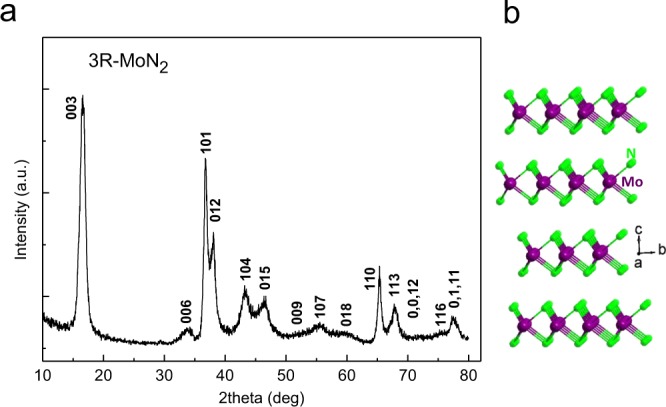
(**a)** X-ray diffraction pattern of rhombohedral 3R-MoN_2_ taken at room temperature. (**b**) Crystal structure of 3R-MoN_2_ characterized by the stacking of N-Mo-N sandwich layers.

**Figure 2:**
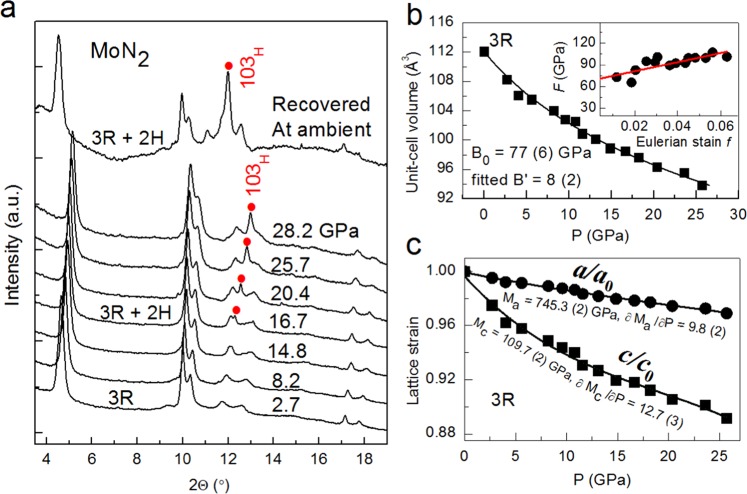
(**a**) Selected high-*P* synchrotron XRD patterns of 3R-MoN_2_ upon room-temperature compression in a DAC. The incident x-ray wavelength (λ) is 0.424603 Å. Red dots denote the new Bragg reflection (103) originated from the hexagonal phase. (**b**) Pressure-volume data of 3R-MoN_2_ fitted to the 3^rd^ Birch-Murnaghan equation of state. The inset shows the normalized pressure (*F*) as a function of Eulerian strain (*f*). (**c)** Calculated linear elastic moduli along *a-* and *c-*axis for 3R-MoN_2_, using the EoSFit program as made by Angel *et al*.^31^. All the error bars in (**b,c**) are too small to be visible.

Figure 1a shows a typical XRD pattern of the purified product synthesized at 3.5 GPa and 753 K for 20 hours through a step reaction. The refined lattice parameters *a* = 2.854 Å and *c* = 15.938 Å agree well with previously reported values^5^. The crystal structure of the rhombohedral 3R-MoN_2_ is shown in Fig. 1b, exhibiting a layered structure similar to that of MoS_2_. Figure 2a shows selected high-*P* synchrotron XRD patterns of 3R-MoN_2_. During room-temperature compression, a new peak around *2Θ* = 13° was observed at ~14.8 GPa, and with further increasing pressure its diffraction intensity increases progressively, indicating the formation of a new MoN_2_ phase (also see Fig. S1). The two MoN_2_ phases coexist up to the highest experimental pressure of 30 GPa. After the release of the pressure, this phase was recovered at ambient conditions. As will be discussed below, this phase is referred to 2H-MoN_2_. Shown in Fig. 2b is the pressure-volume data of 3R-MoN_2_ fitted to the 3^rd^ order Birch-Murnaghan equation of state. In the inset, the normalized pressure (*F*) is plotted vs. the Eulerian strain (*f*). The obtained bulk modulus, *B*_0_, is 77 (6) GPa with *B’* = 8 (2), indicating that MoN_2_ is slightly stiffer than MoS_2_ (*B*_0_ ≈ 53 GPa and *B’* ≈ 9)^24^. This difference is likely due to the enhanced cation-anion bonding in the nitride, because compared with sulfur, the nitrogen is more favorable for the formation of strong covalent bonding states with transition metals. In addition, as shown in Fig. 2c, the *c*-axis is substantially more compressible than the *a*-axis, a behavior that is usually expected in the layer-structured materials. Also noted is that the elastic compressibility of the newly formed 2H phase would be similar due to the structural similarity of both phases as will be discussed below.

**Figure 3:**
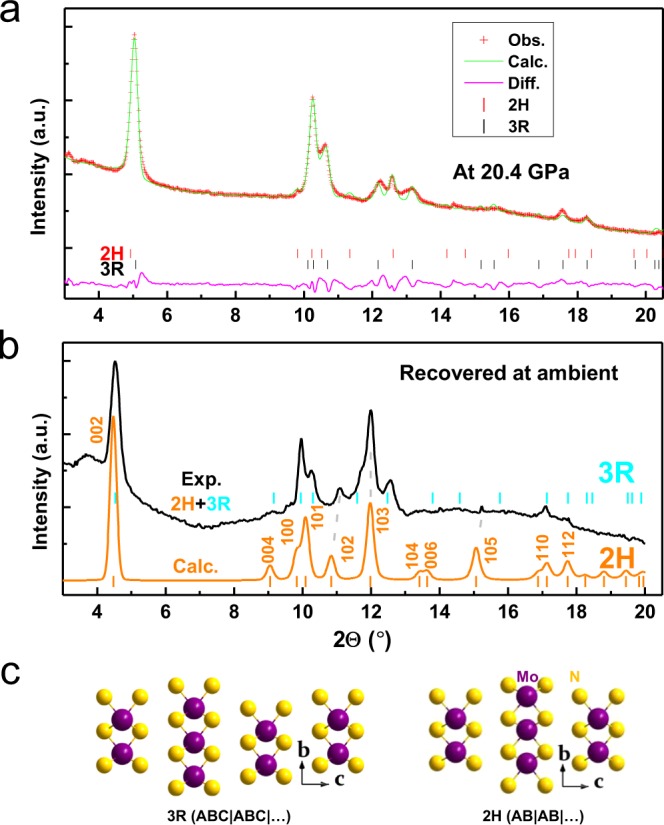
(**a**) Refined XRD pattern for 3R- and 2H-MoN_2_ at 20.4 GPa. (**b)** XRD pattern of the recovered sample with coexistence of 3R and 2H phases. In (**a,b**), the incident x-ray wavelength (λ) is 0.424603 Å. (**c**) Comparison of crystal structures between 3R and 2H polymorphs.

For the newly emerged phase at high pressure, as mentioned above it is well known that the layer-structured MoS_2_ often exists in two different polymorphs: rhombohedral 3R and hexagonal 2H phases; the major difference between them is in the layer stacking sequence^20^. Accordingly, the 2H-MoS_2_-type structure with the space group *P6*_3_*/mmc* was used for analyzing the XRD data of this new MoN_2_ phase. As shown in Fig. 3a, the structure refinement shows that the calculated XRD profile is in excellent agreement with the observed data taken at 20.4 GPa with the coexistence of both rhombohedral and hexagonal polytypes. It is noted that the large background of high-P XRD patterns should originate from the sample crystallinity, because the synthesis of high-quality MoN_2_ sample is still challenging as descripted in our previous report, referring to ref.^5^. This hexagonal phase is therefore referred to as 2H-MoN_2_, and the refined structural parameters are listed in Table 1. The phase transition between 2H- and 3R-MoN_2_ is presumably associated with the rotation and translation of N-Mo-N sandwich layers as previously reported in MoS_2_^20^. Because of their slight structural difference, it is challenging to distinguish between the 2H and 3R phases using the TEM techniques (Fig. S3). It is found that the pressure-induced phase transition from the rhombohedral 3R-MoN_2_ phase to a hexagonal 2H-MoN_2_ structure is irreversible, as referred from the experimental and theoretical x-ray diffraction patterns of MoN_2_ at ambient conditions in Fig. 3b. Also noted is that the density of high-P 2H phase is anomalously lower than that of 3R-MoN_2_ at 20.4 GPa as listed in Table 1. This is not unexpected because the transition from 3R to 2H is often kinetically difficult for achieving a complete conversion; as a result, the final 2H phase would involve a large fraction of the layer stacking disorder and strain in the lattice, which will lead to a significant lattice expansion, hence the reduced the density. For 2H-MoN_2_, compared with simulated XRD pattern (see Fig. 3b), a slight peak shift of the 102 and 105 suggests a severe strain or stress that may build up between the interlayer at relatively low pressure. This is because the 2H phase is metastable phase and trends to transform into 3R phase upon decompression, involving a certain degree of the N-Mo-N interlayer rotation and translation. Compared with the 105 reflection, the 102 peak has a large shift relative to the calculated, probably because of the different stress as induced by interlayer stacking disorder involving different number of layers. The similar phenomenon has been observed in refs^5,20^. As depicted in Fig. 3c, the only crystal structural difference between of 3R- and 2H-MoN_2_ is the interlayer stacking mode. In fact, for most known layer structured materials, they often adopt a common structure of hexagonal 2H polytype (AB|AB|…) with space group of *P*6_3_/*mmc* (No. 194), including TM dichalcogenides TM*X*_2_ (TM = Nb, Mo, Ta, and W; *X* = S and Se). In contrast, the 3R rhombohedral polytype (ABC|ABC|…) is a high-temperature phase with a space group of *R*3*m* (No. 160)^25,26^. Therefore, 3R-MoN_2_ may be a high-T phase with a space group of R3m (No. 160), compared with 2H-MoN_2_ with a space group of P63/mmc (No. 194)^18,25^.

**Table 1:** Summary of structural parameters for MoN_2_ phases determined by analyses of x-ray and neutron diffraction data.

	3R-MoN_2_		2H-MoN_2_
*P-T* conditions	Ambient	20.4 GPa, 300 K	20.4 GPa, 300 K
Formula	Mo_3_N_6_		Mo_2_N_4_
System	rhombohedral		hexagonal
Space group	*R*3m (No. 160)		*P6*_3_/mmc (No. 194)
Cell parameters [Å]	*a* = *b* = 2.854 (1)	*a* = *b* = 2.776 (3)	*a* = *b* = 2.757 (3)
	*c* = 15.938 (2)	*c* = 14.380 (3)	*c* = 9.910 (2)
Cell volume [Å^3^]	112.42 (4)	95.968 (3)	65.235 (2)
Density [g•cm^−3^]	5.492 (2)	6.433 (3)	6.310 (2)
Mo Wyckoff site	Mo1: 3*a*, (0, 0, 0.000 (1))		Mo1: 2*c*, (1/3, 2/3, 1/4)^c^
N Wyckoff sites	N1: 3*a*, (0, 0, 0.258 (1)) N2: 3*a*, (0,0,0.402 (2))		N1: 4 *f*, (1/3, 2/3, 5/8)^d^
*d*_Mo-N_ [Å]	1.976, 2.037		
D_Interlayer_ [Å]^a^	5.313 (1)		
D_Layer distance_ [Å]^b^	3.017 (1)		
*R*_*P*_, w*R*_*P*_ [%] (XRD)^e^	2.1, 3.4	1.7, 2.3	1.7, 2.3
*R*_*P*_, w*R*_*P*_ [%] (NPD)	3.2, 5.3		
*Refs*	ref.^4^	This study	This study

^a,b^D_Interlayer_ is the distances between the two nearest neighboring Mo planes, and D_Layer distance_ corresponds to the distance between the N planes.

^c,d^Proposed atomic positions for 2H-MoN_2_, which cannot be refined accurately using the current high*-P* XRD data.

^e^R_P_ and wR_P_ represent the profile residual and the weighted profile R-factor of refined XRD patterns.

**Figure 4:**
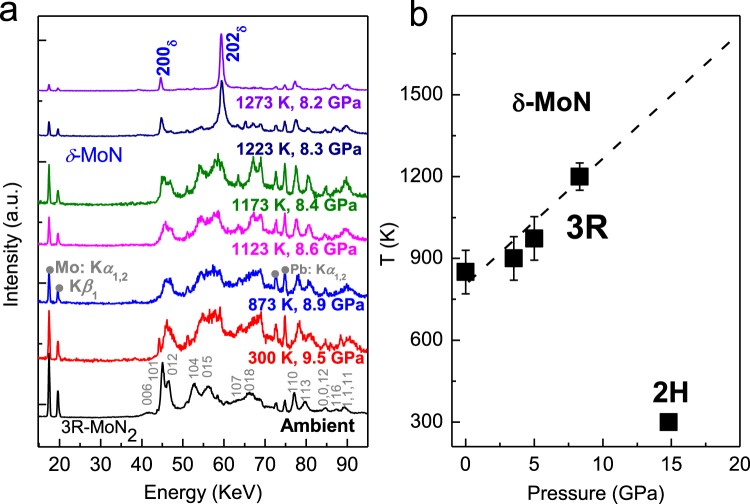
(**a**) *In situ* high P-T energy-dispersive synchrotron XRD measurements. Fluorescence peaks of Mo: K_α,β_ and Pb: K_α_ are denoted by grey solid dots, which originate from MoN_2_ and lead shielding of the detector, respectively. (**b**) Thermal stability of 3R as a function of pressure. δ-MoN forms through the degassing of 3R-MoN2 at high temperature.

To study the phase stability at high temperature, we performed *in-situ* energy-dispersive high *P-T* synchrotron XRD measurement using a large volume pressure. As shown in Fig. 4a, the XRD patterns were collected on heating at a constant load of 80 ton. Because of the thermal effect of the sample cell, the corresponding pressure decreases from 9.5 GPa at 300 K to 8.2 GPa at 1273 K. The strongest Bragg reflection (003) of 3R-MoN_2_ is located at the low-energy region, and it is thus undetectable by the energy-dispersive XRD measurement. Clearly, *δ*-MoN is formed at ~1200 (20) K and 8.3 GPa through nitrogen degassing of 3R-MoN_2_. The decomposition process is expressed by,

1 $${{\rm{MoN}}}_{{\rm{2}}}={\rm{MoN}}+1/2\,{{\rm{N}}}_{{\rm{2}}}$$

The phase stability of 3R-MoN_2_, however, is substantially enhanced with increasing pressure, leading to highly increased N_2_ decomposing temperature (see Fig. 4b). At 8.3 GPa, for example, the reaction (1) happens at a higher temperature of ~1200 K based on *in-situ* high *P-T* synchrotron XRD measurement. The phase diagram of MoN_2_ is eventually determined and summarized in Fig. 4b. Apparently, at high temperature, the *δ-*MoN can be formed through nitrogen degassing of 3R-MoN_2_, on the basis of our previous study of this material in ref.^5^. However, it is experimentally difficult to determine the phase boundary between 2H and 3R at high temperatures above 15 GPa. Nevertheless, the 3R phase seems thermodynamically more stable than 2H phase at high temperature as shown in Fig. 4b. Further experimental work is warranted to determine the detailed phase boundary between 3R and 2H using the state-of-the-art high-P techniques.

**Figure 5:**
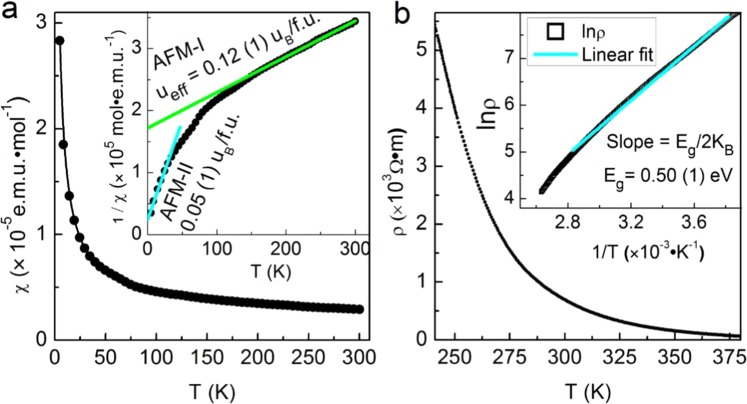
(**a**) Magnetic susceptibility as a function of temperature. The measurements were performed in an external magnetic field of *H* = 1000 Oe. The inset shows the inverse susceptibility, 1/*χ*, against *T*. It seems both AFM phases coexist in the 30–130 K temperature range. (**b**) Four-point probe resistivity as a function of temperature. The measurements were conducted on a well-sintered bulk sample. The inset shows a linear fit of lnρ vs. 1/T based on the expression $\rho ({\rm{T}})={\rho }_{0}{e}^{({E}_{g}/2{k}_{B}T)}$, which yields a band gap of *E*_*g*_ = 0.50 (1) eV.

Figure 5 shows the magnetic and electrical resistivity measurements. At first glance, the low*-T* magnetic susceptibility (χ) data plotted in Fig. 5a suggests a weak paramagnetic-like behavior, as also reported in Li*X*N_2_ (*X* = Mo and W)^27,28^. However, after further analyses, the data can be fitted to the Curie-Weiss law in two temperature ranges, 130–300 K and 2–30 K. The obtained Weiss constants are T_Θ_ = −283 (10) K and −6 (2) K, respectively, indicating that there exist two weakly antiferromagnetic (AFM-I and -II) phases for 3R-MoN_2_. The corresponding magnetic moments of Mo atom are *μ*_*eff*_ = 0.12 (1) and 0.05 (1) *μ*_B_/f.u. Such weak antiferromagnetism in 3R-MoN_2_ presumably originates from the magnetic zigzag edges at the grain boundaries as reported for MoS_2_^29^. The detailed magnetic measurements are listed in Table 2. Moreover, it is worthwhile to mention that the atomically-thin MoN_2_ layers possess intrinsic high-T ferromagnetic properties on the basis of recent *ab-initial* calculations^18^, which may be closely associated with the observed magnetism in this work.

**Table 2:** Magnetic parameters of 3R-MoN_2_ derived from the magnetic susceptibility measurements.

	AFM-I	AFM-II
Temperatures [K]	130–300	2–30
Curies constant, C [10^−3^ emu·mol^−1^·K]	1.69 (1)	0.29 (1)
Weiss constant, T_Θ_ [K]	−283 (10)	−6 (2)
Magnetic moment, *μ*_eff_ [*μ*_B_/f.u.]	0.12 (1)	0.05 (1)

The magnetic susceptibility data were fitted to the Curie-Weiss law χ = C/(T − T_Θ_) in two different temperature ranges of 130–300 K and 2–30 K.

As shown in Fig. 5b, the electrical resistivity of 3R-MoN_2_ increases dramatically as temperature decreases, which is characteristic of a semiconductor. Based on the typical law for a semiconductor, $\rho (T)={\rho }_{0}{e}^{({E}_{g}/2{k}_{B}T)}$, a linear fit of the ln*ρ* - 1/*T* data yields a narrow band gap of *E*_*g*_ = 0.50 (1) eV, which is comparable to the value of 0.47 eV for PbS^30^. However, it is substantially smaller than that of MoS_2_, an indirect semiconductor with *E*_*g*_ ≈ 1.2 eV^29^. In contrast, LiMoN_2_ and LiWN_2_ both behave as an intrinsic metal; the two ternary nitrides also adopt the 3R symmetry of its parent MoN_2_ with intercalated Li layers^27,28^. The measured semiconductor-like behavior in 3R-MoN_2_ may be partially associated with the degrees of crystallinity and defects, such as layer stacking faults. Hence, further resistivity measurements on well-crystallized samples are warranted to clarify this issue.

## Conclusions

In summary, the structural stability and compressibility of a newly discovered layer-structured rhombohedral 3R-MoN_2_ have been studied using high-P compression measurements. A recoverable 2H–MoN_2_, isotypic with hexagonal MoS_2_, is also discovered via high–pressure processing of 3R–MoN_2_, probably involving the interlayer rotation and translation as reported in MoS_2_. Because of their structural similarity, the obtained bulk modulus and axial compressibility for 3R–MoN_2_ are comparable to those of MoS_2_. The obtained the bulk modulus for 3R-MoN_2_ is *B* = 77(6) GPa and the axial compressibility along *c* axis is much softer than that along *a* axis, confirming that the sandwich interlayers are loosely bonded by Van der Waals force. At a high temperature exceeding ~873 K, 3R-MoN_2_ transforms into hexagonal *δ-*MoN through the degassing of N_2_ and this disassociation temperature increases as pressure increases. Besides, 3R-MoN_2_ is weakly antiferromagnetic, may resulting from the occurrence of magnetic zigzag edges in the structure. Moreover, the nitride behaves as a semiconductor with *E*_*g*_ = 0.50(1) eV.

## Supplementary information

Phase Stability and Compressibility of 3R-MoN2 at High Pressure
