# Supplementary material for: Phase Stability and Compressibility of 3R-MoN2 at High Pressure
Source: Sci Rep. 2019 Jul 19;9:10524. doi: 10.1038/s41598-019-46822-4 (PMC6642113; doi:10.1038/s41598-019-46822-4)
Supplement: Supplementary file 1 — Phase Stability and Compressibility of 3R-MoN2 at High Pressure [file 41598_2019_46822_MOESM1_ESM.pdf]

Supporting Information

Phase Stability and Compressibility of 3R-MoN_2 at High Pressure

Xuefeng Zhou,[1] Mingqi Yan,[1] Mingdong Dong,[1] Dejiang Ma,[1] Xiaohui Yu,[2] Jianzhong Zhang,[3] Yusheng Zhao,[1] Shanmin Wang*,[1]

[1]*Department of Physics, Southern University of Science & Technology, Shenzhen 518055, China*

[2]*Institute of Physics, Chinese Academy of Sciences, Beijing 100190, China*

[3]*Materials Science & Technology Division, Los Alamos National Laboratory, Los Alamos, NM 87545, USA*

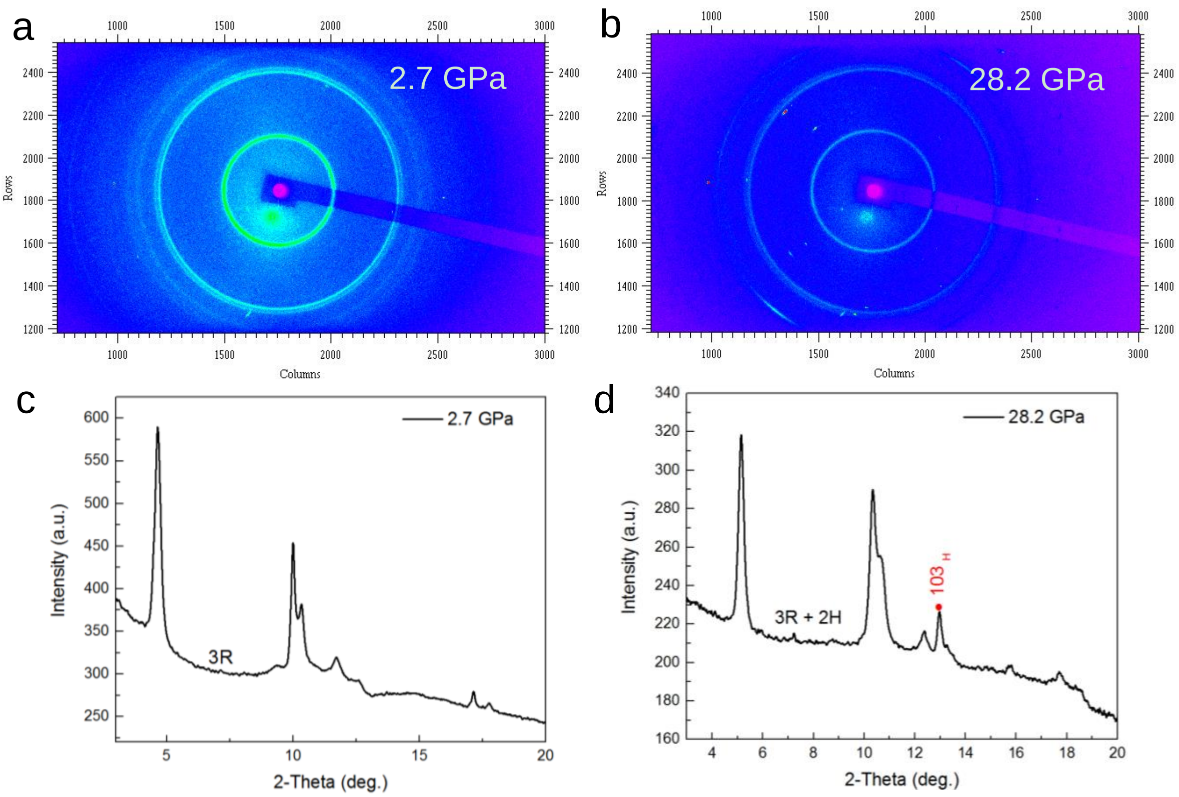

Fig. S1. High-P XRD pattern for MoN_2 taken on compression. (**a**) Bragg diffraction rings for 3R phase collected at 2.7 GPa. (**b**) Bragg diffraction rings for the mixture phase coexisting 3R- and 2H-MoN_2 collected at 28.2 GPa. (**c**) and (**d**) The corresponding two-dimensional diffraction data. The occurrence of texture of 103 in (b) may be associated with the pressure-induced crystal growth or preferred crystallographic orientation.

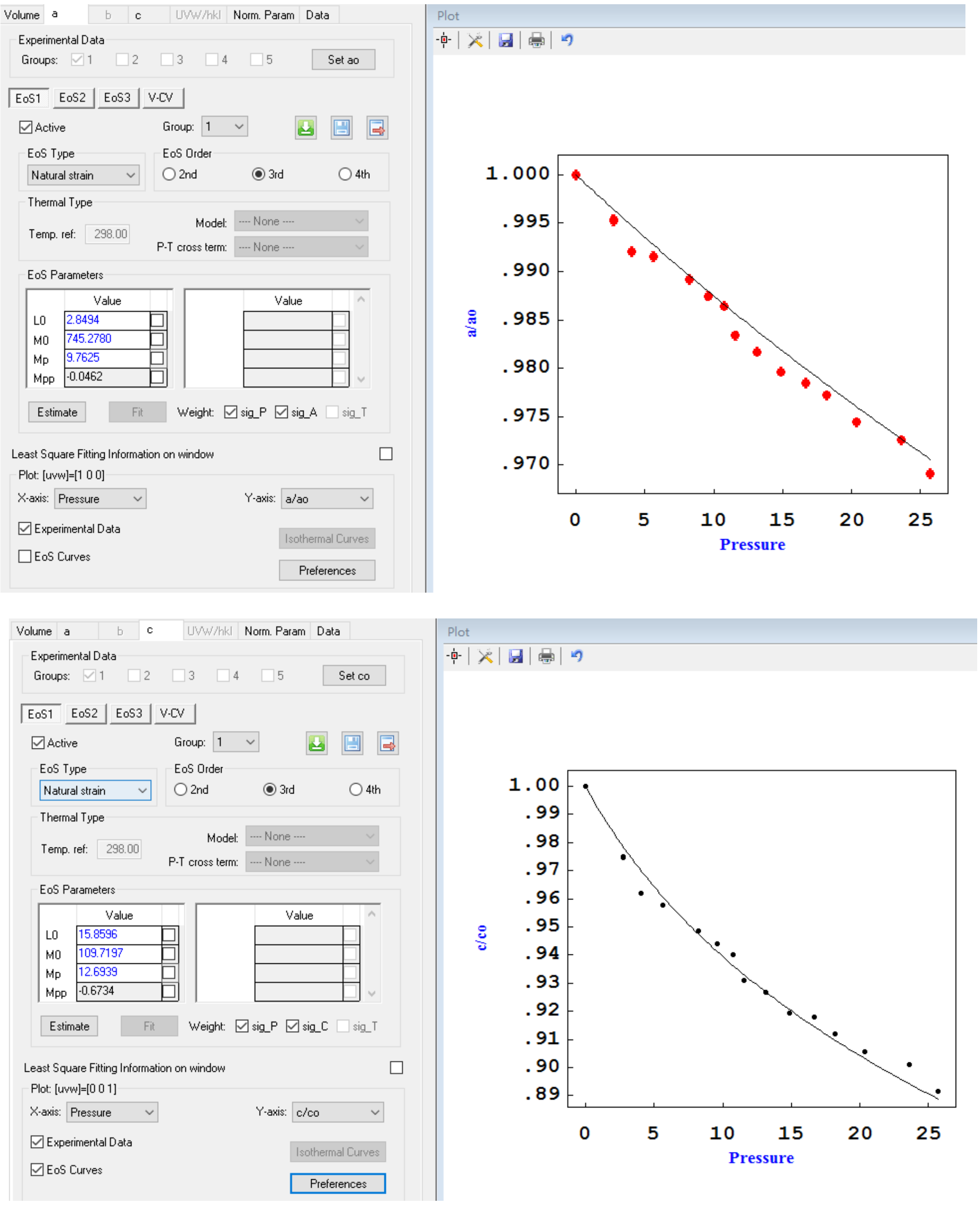

Fig. S2. Snapshots of the fitting process for axial linear modulus along the a- and c-axis using the EosFit programm.[1]

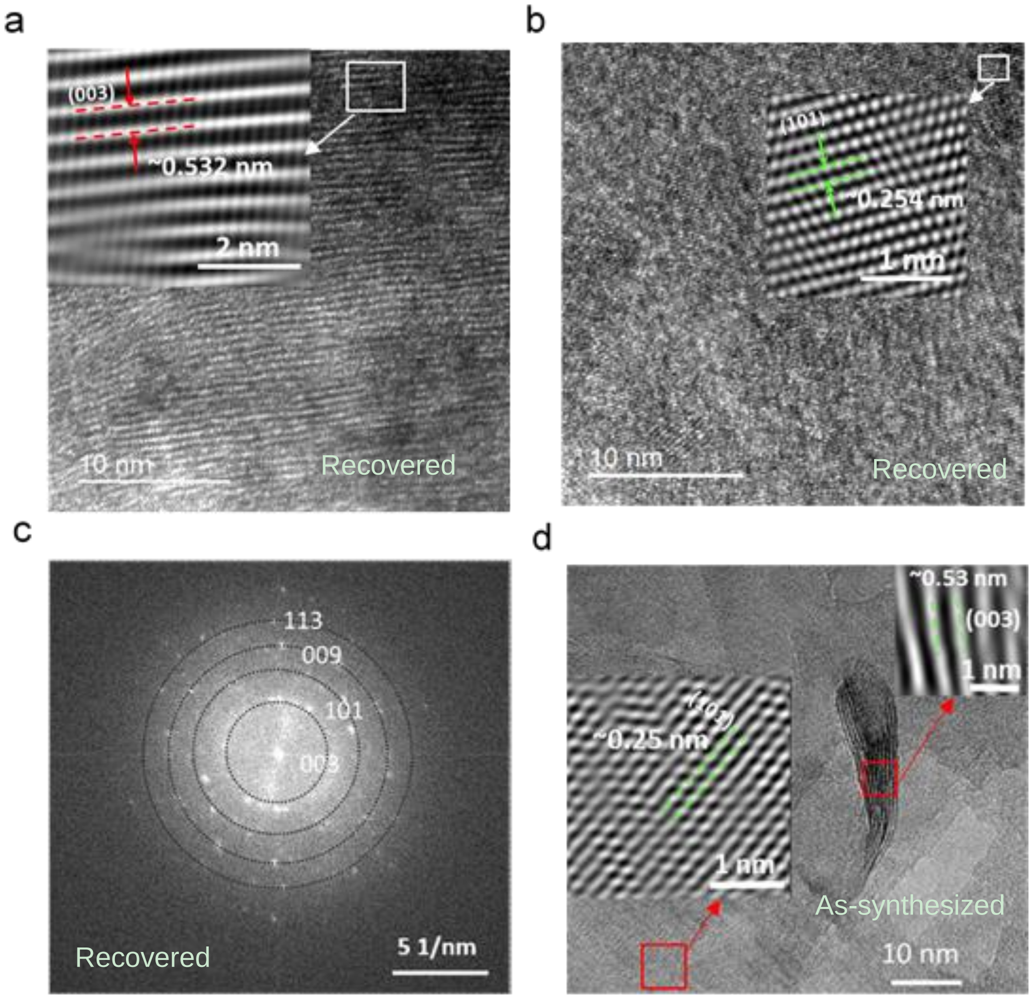

Fig. S3. HRTEM images and electron diffraction patterns for as-synthesized 3R-MoN_2 and recovered from 40 GPa. (**a**) Atomic-scale images of the N-Mo-N interlayers, i.e., the (003) plane, and (**b**) the (101) plane for 3R-MoN_2 recovered from 40 GPa. Insets in both (a) and (b) are the enlarged portions of the regions to show the detail at the atomic level. Dotted double red lines and green lines indicate the d-spacing for the fingerprints of (003) and (101) planes, respectively. (**c**) Selected area electron diffraction, SAED. The observed four diffraction rings correspond to the lattice planes of (003), (101), (009) and (113) for the sample recovered from 40 GPa. (**d**) Atomic-scale images of the N-Mo-N interlayers, i.e., the (003) plane, and the (101) plane for 3R-MoN_2 at ambient. The insets are the enlarged portions of the regions. Dotted double green lines denote

the d–spacing for the fingerprints of (003) and (101) planes, respectively.

References:

1. Angel, R. J., Equations of State. *Rev. Mineral. Geochem.* **2000,** *41* (1), 35-59.
